# Age-Specific Adult Rat Brain MRI Templates and Tissue Probability Maps

**DOI:** 10.3389/fninf.2021.669049

**Published:** 2022-01-07

**Authors:** Eilidh MacNicol, Paul Wright, Eugene Kim, Irene Brusini, Oscar Esteban, Camilla Simmons, Federico E. Turkheimer, Diana Cash

**Affiliations:** ^1^Department of Neuroimaging, Institute of Psychiatry, Psychology & Neuroscience, King's College London, London, United Kingdom; ^2^School of Biomedical Engineering and Imaging Sciences, Faculty of Life Sciences & Medicine, King's College London, London, United Kingdom; ^3^Department of Biomedical Engineering and Health Systems, KTH Royal Institute of Technology, Stockholm, Sweden; ^4^Department of Neurobiology, Care Sciences and Society, Karolinska Institute, Stockholm, Sweden; ^5^Department of Psychology, Stanford University, Stanford, CA, United States; ^6^Department of Radiology, Lausanne University Hospital and University of Lausanne, Lausanne, Switzerland

**Keywords:** template, tissue prior, Sprague-Dawley, preclinical imaging, aging, morphometry, VBM

## Abstract

Age-specific resources in human MRI mitigate processing biases that arise from structural changes across the lifespan. There are fewer age-specific resources for preclinical imaging, and they only represent developmental periods rather than adulthood. Since rats recapitulate many facets of human aging, it was hypothesized that brain volume and each tissue's relative contribution to total brain volume would change with age in the adult rat. Data from a longitudinal study of rats at 3, 5, 11, and 17 months old were used to test this hypothesis. Tissue volume was estimated from high resolution structural images using *a priori* information from tissue probability maps. However, existing tissue probability maps generated inaccurate gray matter probabilities in subcortical structures, particularly the thalamus. To address this issue, gray matter, white matter, and CSF tissue probability maps were generated by combining anatomical and signal intensity information. The effects of age on volumetric estimations were then assessed with mixed-effects models. Results showed that herein estimation of gray matter volumes better matched histological evidence, as compared to existing resources. All tissue volumes increased with age, and the tissue proportions relative to total brain volume varied across adulthood. Consequently, a set of rat brain templates and tissue probability maps from across the adult lifespan is released to expand the preclinical MRI community's fundamental resources.

## 1. Introduction

Animal models of aging are valuable for understanding age-related brain changes. The process of physiological and cognitive decline with increasing age is near-universal in mammals (Morrison and Baxter, 2012); for example, during their lifespan, rodents spontaneously demonstrate age-related cellular changes in regions susceptible to aging in humans (Gallagher and Nicolle, 1993). Researchers can also manipulate the rate or quality of aging with dietary restriction (Carlson and Hoelzel, 1946) or environmental enrichment (Kempermann, 2019), while genetic and environmental confounds can be robustly controlled compared to human studies. Crucially, this control over experimental settings affords findings which are expected to generalize across species (e.g., to humans) when using homologous methods.

One such method is MRI: in theory, its non-invasive application is equivalent in humans and other animals. However, human brain MRI is a more mature field than its preclinical equivalent (i.e., MRI of experimental animals); hence the latter has fewer foundational resources and software tools. For example, popular neuroimaging toolboxes such as SPM (Ashburner et al., 2014) or FSL (Jenkinson et al., 2012) assume human MRI data as input and rely heavily on prior information. Prior information can take the form of, e.g., reference images, which are often the average of a representative sample and known as *templates*; and tissue probability maps (TPMs), which use probabilistic classifications of voxels to extract biologically meaningful classes. Such information is population-specific and does not generalize across species. For instance, compared to human brains, rodent brains are elongated along the coronal axis, have a smaller proportion of white matter (WM), and do not have cortical folding.

Species-specific resources, including templates and TPMs, facilitate translation of optimized human imaging protocols to preclinical imaging, but age-specific adult rat resources are not yet available. Human brain morphology is known to change with age (Raz and Rodrigue, 2006), so template choice is non-trivial. For example, registering a child's MR image to an adult template induces inter-subject variability (Fonov et al., 2009), while population-specific templates reduce such bias in studies of older adults (Huang et al., 2010; Fillmore et al., 2015). Age-specific rat MRI resources are available for developmental studies until postnatal day 80 (Calabrese et al., 2013; Rumple et al., 2013), which is unsurprising given the accelerated change within this period. However, there are no rat MRI resources available to reflect age-specific brain changes that likely occur in adulthood and old age. Moreover, only three of the adult rat templates have associated TPMs. Valdés-Hernández et al. (2011) generated the first set by assigning tissue class from the image intensity distribution, which incorrectly classifies the thalamus as WM. The SIGMA set (Barriere et al., 2019) was derived from the Valdés-Hernández et al. (2011) TPMs and, although the template was improved with respect to field of view and contrast-to-noise ratio, the thalamic error in the TPMs persisted. The most recent set (Goerzen et al., 2020) applied an atlas-based approach that rectifies this error, but it is derived from a less commonly used Fischer344 strain. As the structural similarity between laboratory rat strains is unknown, application to other strains requires validation.

This report addresses two aims: first, can classification of thalamic gray matter (GM) be improved for Sprague-Dawley rats by creating new adult rat MRI resources? New TPMs were generated by combining anatomical prior knowledge and signal intensity information, rather than relying purely based on signal intensity. The resulting TPMs were compared to existing resources by extracting GM volume (GMV) estimates from a thalamic region of interest (ROI) and to histological sections of an adult rat thalamus. The second aim was to test if the hypothesized volumetric changes occur in a rat model of aging. Total intracranial volume (TIV) and its constituent tissue ratios were estimated from a longitudinal study of the adult lifespan. The resources detailed herein have been openly released, equipping researchers with reliable prior knowledge to integrate into their workflows.

## 2. Methods and Materials

### 2.1. Animals

Animal experiments were in accordance with the UK Home Office Animals (Scientific Procedures) Act (1986) and were approved by the King's College London ethical review committee. The RESILIENT study followed male Sprague-Dawley rats (*N* = 72) across a maximum of four scanning sessions at 3, 5, 11, and 17 months old, which approximately correspond to late adolescence, young adulthood, middle age, and the beginning of senescence (Quinn, 2005). Rats (Charles River, UK) were received at 1-month-old ± 4 days and group-housed under standard light (12:12 light:dark), temperature (21 ± 2°C), and humidity (55 ± 15%) conditions with free access to water. At each session, the rats underwent a battery of behavior tests and a comprehensive MRI protocol detailed elsewhere (MacNicol, 2021). Exclusion criteria included presenting with health problems (*N* = 13; e.g., weight more than 850 g, diabetes, arthritis, or tumor growth) or if rats could not be socially-housed (*N* = 12) since isolation was expected to be a confound. One rat was excluded after data acquisition due to a latent irregular brain mass.

The RESILIENT study primarily aimed to assess lifestyle modifications in aging, so a group (*N* = 24) was subjected to life-long environmental enrichment and dietary restriction (EEDR) after the first scan, while the remaining rats served as controls for *resource generation* (*N* = 24) and *testing* (*N* = 24). The EEDR treatment and its effects are discussed elsewhere (MacNicol, 2021). Briefly, environments were enriched with extra toys and food was removed on three non-consecutive days a week for 24 h, with *ad libitum* access to standard chow on non-fasting days. Control rats were kept in standard caging, enriched with a cardboard tube and wooden chewsticks, with *ad libitum* access to standard chow. However, relevant to the current report, the RESILIENT cohort showed more heterogeneous aging outcomes than those observed in standard cohorts of laboratory rats, and hence are relevant to a multitude of rat models. Based on the exclusion criteria and groups described here, the results presented are based on the following sample sizes at each session: *N*_*EEDR*_ = 24, 24, 23, 21; *N*_*resource*_ = 24, 24, 16, 9; *N*_*testing*_ = 24, 22, 17, 9.

### 2.2. Image Acquisition

Anesthesia was induced with 5% isoflurane in a 30:70 mixture of oxygen in medical air at 1.4 liters per minute and maintained on 2–2.5% isoflurane during scanning (2–3 h). Body temperature was maintained at 37 ± 1^*o*^C using an embedded water heating system and thermostatically controlled hot air paired to a rectal thermometer. Physiology monitoring tools (thermometer, pulse oximeter, and respiration balloon) were made by Small Animal Instruments, Inc., NY, USA. The rats were scanned in a 9.4 T Bruker Biospec MR scanner with an 86-mm volume coil for transmission and a four-channel array receiver coil. Protocols were implemented using Paravision 6.0.1 (Bruker Corp., Ettlingen, Germany). High-resolution anatomical images were acquired with a 3D MP2RAGE sequence: repetition time (TR) = 9,000 ms; inversion times (TIs) = 900, 3,500 ms; flip angle = 7^*o*^, 9^*o*^; echo time (TE) = 2.695 ms; echo TR = 7.025 ms; matrix = 160 × 160 × 128; and 0.19 mm isotropic voxel size. A 3D ultra-short echo (UTE) reference scan was also acquired with the following parameters: TE = 8 μs, TR = 3.75 ms, flip angle = 4^*o*^, matrix = 128 × 128 × 128, and 0.45 mm isotropic voxel size.

### 2.3. Image Preprocessing

The MP2RAGE images were processed using QUantitative Imaging Tools v2.0.2 (Wood, 2018). The complex image from each coil channel was combined using the UTE reference image and the COMPOSER method (Robinson et al., 2017). The combined image was input to qi_mp2rage, which uses the signal at both inversion times to produce a *T*_1_ map and a *T*_1_-weighted (T1w) image that is inherently corrected for *B*_1_ field inhomogeneity, which causes non-uniformity of the signal intensity across an image.

### 2.4. Resource Generation

**Figure 1:**
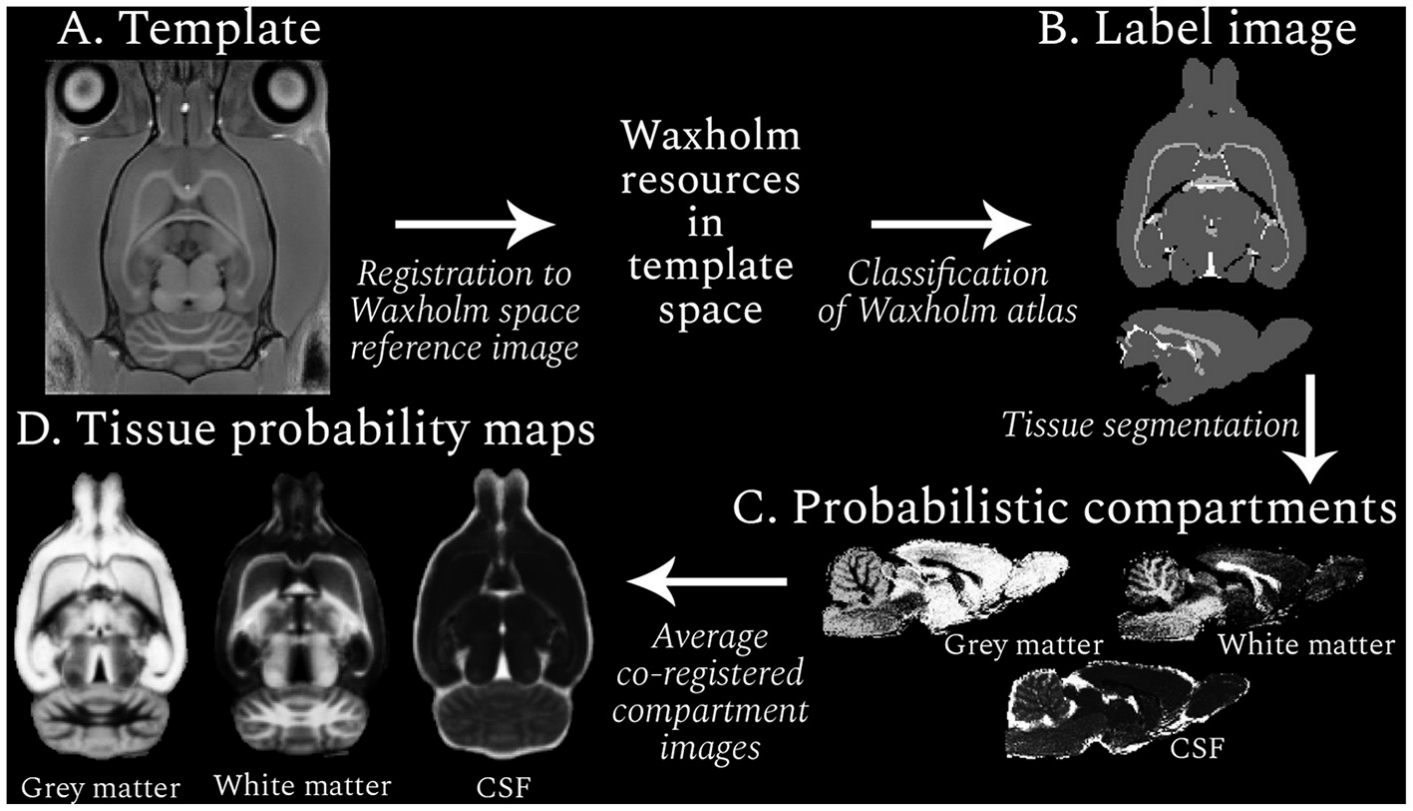
Resource generation workflow (clockwise from top left). An example RESILIENT template is shown **(A)**, which was registered to the Waxholm space reference image to facilitate use of the associated resources. Regions in the Waxholm space atlas were designated as one of three tissue types, and the resulting label image **(B)** was used to generate probabilistic compartment images for each subject **(C)**. The co-registered compartments were averaged across subjects to produce tissue probability maps **(D)**. The voxel intensity in the probabilistic images represents the likelihood, from 0 (black) to 1 (white), of the presence of a given tissue.

*Resource generation* control and EEDR T1w images within a session were co-registered to create four age-specific, average templates (Figure 1A) using ANTs (Avants et al., 2010). Each template was normalized to the Waxholm reference (Papp et al., 2014), an *ex vivo* MR image from a Sprague-Dawley rat, using antsRegistration (Avants et al., 2011a), facilitating the projection of the Waxholm atlas to the age-specific template space. Non-brain tissue was removed with a modified implementation of artsBrainExtraction, a rodent-specific atlas-based algorithm (MacNicol et al., 2021) developed for *T*_2_-weighted (T2w) images. The Waxholm reference was the target instead of the default Fischer344 template, and redundant steps for MP2RAGE images (denoising and inhomogeneity correction) were omitted. Brain-extracted T1w images were normalized to the appropriate brain-extracted session template. Jacobian determinant maps, which quantify the voxelwise volume changes resulting from the spatial normalization to the template, were calculated for each individual's transformation.

Regions from the Waxholm atlas third edition (Osen et al., 2019) were categorized as GM (1), WM (2), or cerebrospinal fluid (CSF; 3). To reduce the impact of partial volume effects, regions with non-homogeneous tissue compositions were excluded. The resulting mapping between regions and tissue types provided a tissue type atlas (or *label image*; Figure 1B) that was projected to the subject-space. Voxels within a brain mask were segmented into three compartments with ANTs Atropos (Avants et al., 2011b), leveraging information from the label and T1w images. Thus tissue probability values were propagated to regions not covered by the label image but within the mask, generating probabilistic compartment images per subject for each session (Figure 1C). Notably, the prior weighting was minimized so the label image was used for the initial Gaussian mixture model and the image intensity information was used until the segmentation reached convergence. Compartment images were qualitatively assessed before transformation to the session template and averaging across subjects to create age-specific TPMs (Figure 1D).

### 2.5. Histology

**Figure 2:**
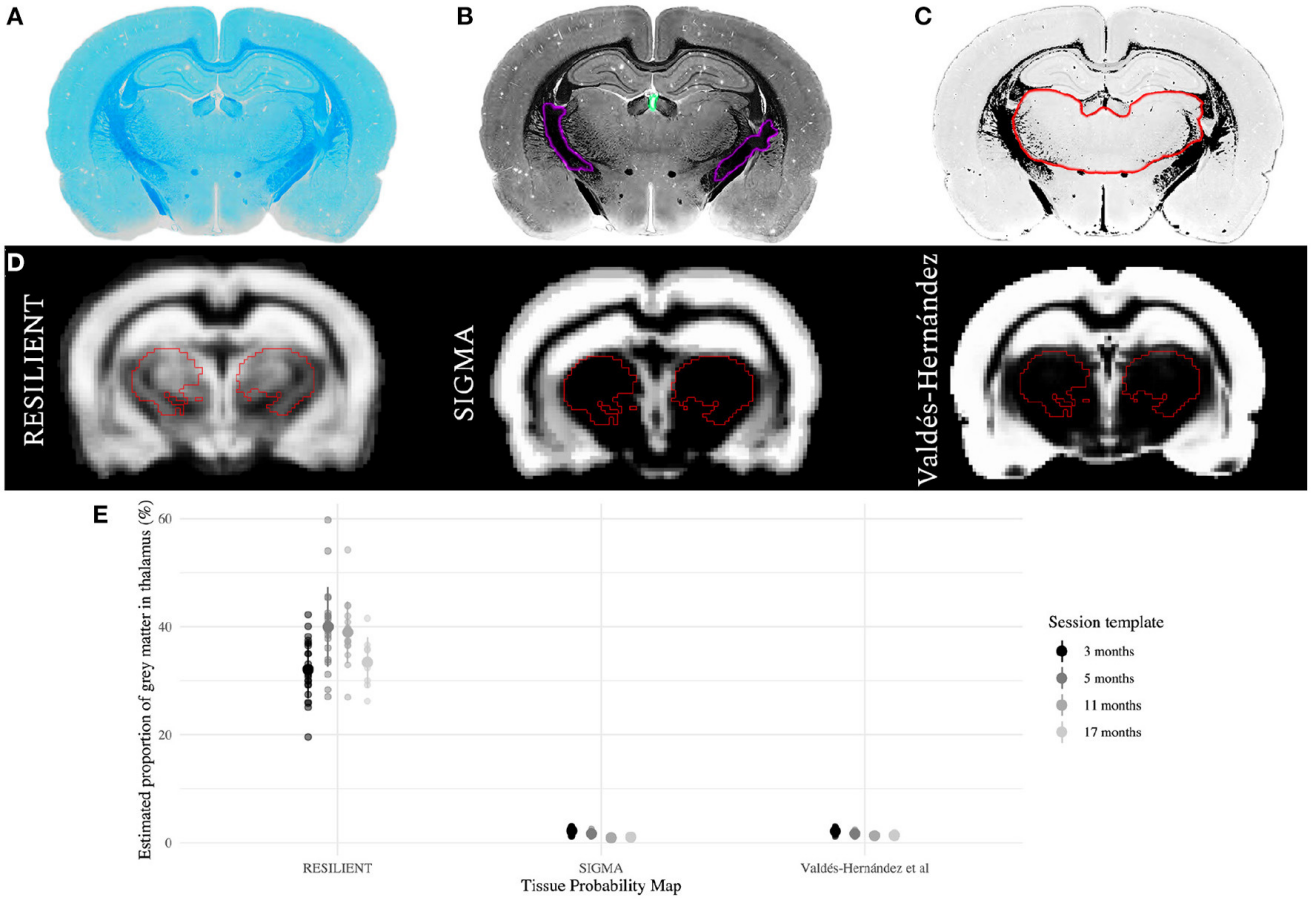
Comparison of thalamic gray matter (GM) estimates. **(A–C)** Qualitative histological estimation of thalamic GM. A section stained for myelin with Luxol Fast Blue **(A)** was transformed to gray-scale **(B)**, and the mean ($\bar{X}$) and standard deviation (SD) of pixel intensities were calculated within manually-delineated white matter (WM; purple) and ventricular (turquoise) regions of interest (ROI). The section was binarized **(C)** so that pixels which likely represent GM were valued 1 (white), while all others were valued 0 (black). The hand-delineated thalamus ROI (red overlay) is predominantly GM. **(D)** The RESILIENT TPM has higher prior probabilities of GM within the thalamic ROI (red overlay), derived from the SIGMA atlas, compared with existing resources. **(E)** GM estimates as a proportion of thalamic volume for each TPM set. The mean ± SD of estimates are shown for each set.

A control rat was euthanized for histological processing by transcardiac perfusion with heparinized saline, followed by 4% paraformaldehyde. Brain tissue was cryoprotected in 30% sucrose before sectioning at 35 μm on a freezing microtome. Luxol fast blue (LFB) staining was performed on sections mounted onto microscope slides, as previously described (Wood et al., 2016). LFB stained sections were scanned with an Olympus VS120 slide scanner at x40 magnification (Figure 2A). Each section image was transformed to gray-scale for manual delineation of WM and ventricular ROIs by an expert (author DC). The mean ($\bar{X}$) and standard deviation (SD) of pixel intensities were calculated from each ROI (Figure 2B). Pixels that were likely to represent GM, defined as intensities4 between ${\bar{X}}_{{\text{WM}}^{+}}$ 3 SD and ${\bar{X}}_{{\text{ventricle}}^{-}}$ 3 SD, were binarized so pixel intensities within this window were valued 1, while all pixels were valued 0 (Figure 2C).

### 2.6. Analysis

#### 2.6.1. Thalamic Gray Matter Volume Estimates

The age-specific templates were normalized to the SIGMA and Valdés-Hernández templates using antsRegistration, and the TPMs were projected to each RESILIENT session space. Brain-extracted images from the *testing* controls were transformed to the appropriate age-specific template and segmented with each TPM set using Atropos with identical parameters. The resulting compartment images were multiplied with the Jacobian determinant maps to preserve voxelwise tissue volume (e.g., GMV) information. The bilateral thalamus binary mask, created from the SIGMA anatomical atlas and projected to each age-specific template space, is overlaid on the GM TPMs in Figure 2D. Jacobian-modulated GM compartment values within the mask were summed and multiplied by the voxel volume. The Jacobian determinants within a subject's thalamic mask were summed to report the thalamic volume fraction.

#### 2.6.2. Tissue Volume and Proportional Estimates

Tissue volume estimates were retrieved from the EEDR and *resource generation* groups to test if brain volumes change with age in the adult rat. The first session template was used as a common space since some subjects did not have images from later sessions. Thus, within-subject transformations were generated from non-linear registration of T1w images from consecutive sessions. Each T1w image was projected to the first session template by concatenating the within-subject and subject-to-template transformations before segmentation with the first session TPMs using Atropos. GM, WM, and CSF volumes were estimated by multiplying voxel volume and the sum of voxel values within a brain mask of the corresponding Jacobian-modulated compartment image. The volume estimates were normalized to each subject's TIV, defined as the sum of all three compartments, to test if brain volume proportions vary in adulthood.

#### 2.6.3. Statistics

Linear mixed-effects models were fit with R v3.6.2 using lme4 v1.1-23 (Bates et al., 2015) and lmerTest v3.1-2 (Kuznetsova et al., 2017) packages. Thalamic GMV estimates were predicted by the fixed effects of session and TPM set, while also controlling for the subjects' random effects. GM, WM, CSF, and total intracranial volumes were predicted from age using separate models with session as the fixed effect and controlling for the random effects of subject. Proportions were predicted by the fixed effects of session, tissue type, and the interaction between them, while also controlling for the random effect of subjects. Model estimates are reported as β (or Δ_β_) ± standard error. Estimated marginal means (EMMs) were calculated using emmeans v1.4.7 (Lenth, 2020) and consecutive sessions' estimates were compared. Reported p-values were adjusted for multiple comparisons using a multivariate t-distribution.

## 3. Results

### 3.1. Release of Prior Information for Rat Brains in Their Adulthood

The RESILIENT templates and associated resources are available under a Creative Commons Attribution 4.0 license from the open science framework (osf.io/U4GTW) and *TemplateFlow* (Ciric et al., 2021), a version-controlled repository that faciliates template integration for processing pipelines.

### 3.2. RESILIENT-Derived Thalamic Gray Matter Estimates Differ From Existing Resources

Thalamic GM estimates derived from the RESILIENT TPMs had greater means and standard deviations than those from either existing TPM (Figure 2E). The RESILIENT intercept (35.25 ± 0.60%), representing the 3 month estimate, was higher than either existing TPM intercepts (β_RESILIENT_ - β_VH_ = 34.57 ± 0.64%;β_RESILIENT_ - β_SIGMA_ = 34.68 ± 0.64%; both *p* < 0.0001). The RESILIENT estimates also varied by session: the estimates from the 5 and 11 month TPMs were higher than at 3 months (β_5_ - β_3_ = 2.34 ± 0.65%, *p* = 0.0004; β_11_ - β_3_ = 1.60 ± 0.70%, *p* = 0.0246). There was no significant difference between the first and final session estimates (β_17_ - β_3_ = 0.04 ± 0.88%, *p* = 0.9677).

### 3.3. Brain Volumes Change Across Adulthood in Rats

**Figure 3:**
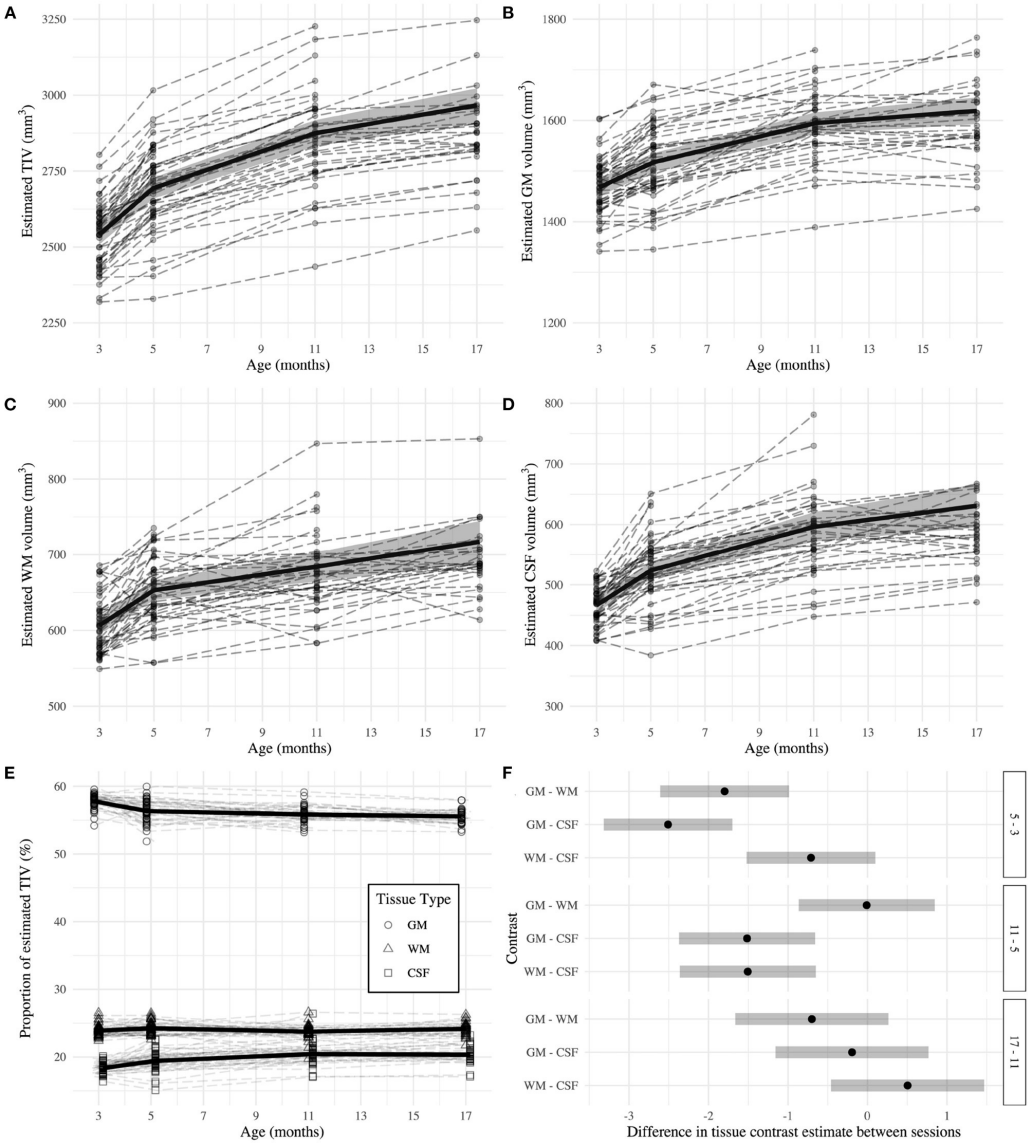
Estimates of total intracranial **(A)**, gray matter **(B)**, white matter **(C)**, and CSF **(D)** volumes across adulthood in the rat. **(E)** Relative proportion of tissue volumes with respect to total intracranial volume. The dashed lines represent individual trajectories, while the solid line and background fill connects the mixed-effects model estimated marginal means ± 95% confidence interval of the estimate. **(F)** Second level contrast plots indicating the size and direction of the difference between contrasts of consecutive sessions for each tissue type. The dots denote the estimate difference and the bars denote the 95% confidence interval of the estimate.

TIV (Figure 3A) was estimated as 2540.98 ± 16.36 mm^3^ at 3 months and the later estimates were greater than the first (β_5_ - β_3_ = 152.77 ± 4.11 mm^3^; β_11_ - β_3_ = 333.44 ± 11.38 mm^3^; β_17_ - β_3_ = 425.43 ± 19.59 mm^3^; all *p* < 0.0001). The GM estimate (Figure 3B) at 3 months was 1467.95 ± 8.77 mm^3^, and subsequently increased (β_5_ - β_3_ = 49.20 ± 2.59 mm^3^; β_11_ - β_3_ = 126.62 ± 33.62 mm^3^; β_17_ - β_3_ = 151.14 ± 8.72 mm^3^; all *p* < 0.0001). WM volume (Figure 3C) was estimated as 606.69 ± 5.85 mm^3^ at 3 months and thereafter increased (β_5_ - β_3_ = 46.18 ± 2.56 mm^3^; β_11_ - β_3_ = 77.32 ± 6.35 mm^3^; β_17_ - β_3_ = 110.46 ± 10.82 mm^3^; all *p* < 0.0001). CSF volume (Figure 3D) was estimated as 466.34 ± 6.02 mm^3^ at 3 months and increased in later sessions (β_5_ - β_3_ = 57.39 ± 2.81 mm^3^; β_11_ - β_3_ = 129.85 ± 8.29 mm^3^; β_17_ - β_3_ = 164.63 ± 14.31 mm^3^; all *p* < 0.0001). The EMMs (Table 1) consecutively increased in all tissue types.

**Table 1:** Consecutive comparison of the Estimated Marginal Means (EMMs) of the linear mixed-effects models.

**Model**	**Tissue**	**Contrast**	**Δ_*EMM*_**	**SE**	**df**	***P*-value**
**Volume (mm** ^**3**^ **)**	TIV	5–3	152.77	4.11	197.95	0.0000
		11–5	180.67	9.12	57.66	0.0000
		17–11	91.99	9.42	68.16	0.0000
	Gray matter	5–3	49.19	2.59	391.91	0.0000
		11–5	77.43	4.43	75.22	0.0000
		17–11	24.52	4.82	103.13	0.0000
	White matter	5–3	46.18	2.56	272.82	0.0000
		11–5	31.14	5.16	64.04	0.0000
		17–11	33.14	5.42	79.88	0.0000
	CSF	5–3	57.39	2.81	160.56	0.0000
		11–5	72.46	6.55	57.70	0.0000
		17–11	34.78	6.72	65.24	0.0000
**Volume/TIV (%)**	GM	5–3	−1.44	0.24	435.74	0.0000
		11–5	−0.51	0.26	446.47	0.1274
		17–11	−0.30	0.29	444.52	0.6175
	WM	5–3	0.36	0.24	435.74	0.3253
		11–5	−0.50	0.26	446.48	0.1377
		17–11	0.40	0.29	444.52	0.3827
	CSF	5–3	1.07	0.24	435.74	0.0000
		11–5	1.01	0.26	446.48	0.0003
		17–11	−0.10	0.29	444.52	0.9712

*The difference in mm^3^ is reported for the volume models, while the percentage change relative to the total intracranial volume (TIV) is reported for the proportional model. Δ_EMM_ denotes the difference in EMMs from the two sessions in the contrast column. SE, standard error. df, degrees of freedom*.

### 3.4. Tissue Proportions Change Across Adulthood in Rats

GM was estimated as 57.79 ± 0.17% of TIV at 3 months, which was higher than the estimate for WM and CSF (β_GM_ - β_WM_ = 33.92 ± 0.24%; β_GM_ - β_CSF_ = 39.44 ± 0.25%; both *p* < 0.0001). GM proportion decreased compared to baseline at subsequent sessions (β_5_ - β_3_ = −1.44 ± 0.24%; β_11_ - β_3_ = −1.94 ± 0.26%; β_17_ - β_3_ = −2.24 ± 0.28%; all *p* < 0.0001). However, each tissue had a distinct relationship with age (Figure 3E). The change in WM proportion from baseline was greater than the same difference in GM proportion (β_*WM*:5−3_ - β_*GM*:5−3_ = 1.80 ± 0.34%; β_*WM*:11−3_ - β_*GM*:11−3_ = 1.81 ± 0.36%; β_*WM*:17−3_ - β_*GM*:17−3_ = 2.51 ± 0.39%; all *p* < 0.0001). The difference from baseline CSF proportion was also greater than the same difference in GM proportion for all comparisons (β_*CSF*:5−3_ - β_*GM*:5−3_ = 2.51 ± 0.34%; β_*CSF*:11−3_ - β_*GM*:11−3_ = 4.02 ± 0.36%; β_*CSF*:17−3_ - β_*GM*:17−3_ = 4.23 ± 0.39%; all *p* < 0.0001).

From 3 to 5 months, GM proportion decreased while CSF proportion increased (Table 1). CSF proportion further increased between 5 and 11 months. There were no significant differences in WM proportion between any consecutive sessions. Figure 3F demonstrates tissue type differences for consecutive session contrasts. A difference of zero suggests both proportions change at approximately the same rate and direction. Alternatively, positive differences indicate the tissue proportions diverge between sessions, and negative differences indicate convergence. First, GM proportion decreases at the expense of WM and CSF. Between 5 and 11 months, the CSF proportion continues to increase while GM and WM proportions plateau at a similar rate. Between 11 and 17 months, there are no differences in proportional change.

## 4. Discussion

Age-specific adult rat MRI resources are released for two ends: to improve thalamic GM estimates and to satisfy a shortcoming in the existing resources, since brain volumes were found to change across adulthood.

### 4.1. RESILIENT Tissue Probability Maps Give Higher Thalamic Gray Matter Estimates

The Valdés-Hernández and SIGMA TPMs estimate thalamic GM proportion between 0.9 and 2.2%, which is very low compared to qualitative histological evidence of myelin staining (Figure 2C), likely caused by minimal GM prior probability in subcortical regions (Figure 2D). In contrast, the RESILIENT TPMs estimate higher thalamic GM proportions, with mean estimates ranging from 30 to 40%. Previous resources relied on histogram thresholding methods to assign tissue classifications, which is fast but problematic for regions with non-homogeneous tissue distribution. Instead, the RESILIENT TPMs provided prior tissue class information by augmenting a popular atlas. However, the RESILIENT estimates still underestimate compared to the qualitative observation. Segmentation with atlas-based TPMs, applied here and by Goerzen et al. (2020), is more accurate than thresholding methods but do not entirely overcome partial volume effects. Voxelwise assignments are inferior to histological validation so future work must test the reliability of atlas-based TPMs based on a ground truth. For instance, could estimating cortical and subcortical GM as separate classes improve segmentation? Nonetheless, by leveraging atlas information and averaging across a large sample, these TPMs provide a favorable approximation of thalamic GM.

### 4.2. Brain Volumes and Tissue Proportions Change With Age in the Adult Rat

All tissue volumes increased with age but their relative proportion of TIV showed distinct trajectories. For instance, in young adulthood, WM has its largest volume increase but does not change proportion. Conversely, GM and CSF proportions change rapidly from 3 to 5 months, but their largest increase is from 5 to 11 months when proportional changes are slowing down. All brain volumes increase between 11 and 17 months, but the proportions stabilize. While an inverse relationship between GM and CSF proportions is also observed in humans (Narvacan et al., 2017), human GM volume decreases from adolescence and regional WM volume declines begin after middle age (Raz et al., 2005). No volume declines were observed here, but the proportion of GM did decrease from adolescence before plateauing.

Inter-species differences or the study design could explain the observed results. In male young adult humans, GM, WM, and CSF occupy approximately 55, 30, and 15% of TIV, respectively (Taki et al., 2011). In male young adult rats, we estimated marginal mean proportions of 58, 24, and 18%. However, these values are ostensibly still impacted by the thalamic GM underestimation described in the previous section, suggesting the ground truth has even more GM and less WM than our findings. Additionally, GM and WM proportions have been found to decrease ~4 and ~1% respectively in male humans between 20 and 50 years old (Fotenos et al., 2005) but here, GM proportions decreased ~2% between 3 and 17 months and WM decreased ~0.5% between 5 and 11 months. However, the window of structural decline was possibly omitted if the study's conclusion was premature. We estimated that the human equivalent age for the final session, 17 months, would be approximately 60 years old but providing an accurate translation between life stages in humans and rats is challenging; only approximations are provided and the relative duration of life stages differ (Quinn, 2005). Human brain volumes seemingly follow an inverted-U trajectory, reaching peak volume after 50 (Raz et al., 2005). As volume increases slowed from 11 to 17 months, perhaps a longer study window would have captured such a trajectory. For example, 27-month old rats had smaller medial prefrontal cortex, striatal, and hippocampal volumes, and larger ventricles than 14-month old rats (Hamezah et al., 2017). However, these differences were observed in a cross-sectional study which could be confounded by latent cohort effects.

The longitudinal study design used here facilitated the recording of individual brain volume trajectories, but sample size retention is a challenging weakness of longitudinal designs. We addressed these issues using linear mixed-effects models, which are more robust to missing data, but the variability at later sessions could have been reduced with larger cohorts. Another limitation of the present study is that it only included males. Sex differences in age-related declines have been observed in clinically normal human populations (McCarrey et al., 2016), so it is important to study a rodent cohort containing both sexes in the future. Despite its shortcomings, these findings justify further investigation of template choices, particularly for preclinical models of aging. For example, do age-specific templates minimize processing biases in registration quality, or is there an optimal age that is best suited as a registration target?

### 4.3. Reliable Prior Information for Translational Protocols

This study's anatomical reference used an MP2RAGE sequence, providing both a T1w image and a *T*_1_ map from one sequence, maximizing the output for a given scan. Further benefits of MP2RAGE sequences include the redundancy of intensity non-uniformity correction, since MP2RAGE sequences are robust to *B*_1_ field inhomogeneity (Marques et al., 2010), and the sensitivity of *T*_1_ to tissue myelin content (Lutti et al., 2014), which facilitates differentiation of WM from GM. Thus the quantitative *T*_1_ values, and the T1w values that have a monotonic mapping to the quantitative values, obtained from an MP2RAGE sequence may be more closely related to the underlying tissue composition than image intensity values from other T1w or T2w image protocols and using MP2RAGE-derived T1w images for TPM generation and tissue segmentation has generated results that reliably represent the underlying tissue.

We intend to encourage the inclusion of MP2RAGE sequences in preclinical imaging protocols. T1w images are chosen for human MRI segmentation studies because they provide good tissue contrast in a short acquisition time. However, T2w anatomical images are used more frequently in studies involving small animals due to image contrast differences at high field strength and a lesser proportion of WM in rodent brains. Thus, we release complementary MP2RAGE-derived *T*_1_ map templates in addition to the T1w templates. Since the spatial distribution of *T*_1_ map values is similar to that of T2w images (i.e., CSF has high, WM has low, and GM has intermediate values), we suggest these can be used as pseudo-T2w templates.

### 4.4. Summary

We deliver MRI resources for the preclinical community, including TPMs that provide improved thalamic GM estimates. Since TPMs are used beyond volume estimates (e.g., in functional MRI denoising), they are necessary for integrating small-animal data into processing workflows designed for human data. We also found age-related changes in brain volume and tissue proportions, relative to TIV, in adult rats. Thus, registration templates for aging studies may require further consideration. Biases from age-related brain compositional changes in human studies are mitigated by using age-specific templates, so we release a set of rat brain MRI templates and TPMs from across adulthood, augmenting preclinical MRI resources.

## Data Availability Statement

The datasets presented in this study can be found in online repositories. The names of the repository/repositories and accession number(s) can be found below: the resources generated for this study can be found in a Open Science Framework repository (10.17605/OSF.IO/U4GTW). Image-derived data (i.e., volume estimates) can be found in a separate OSF repository (10.17605/OSF.IO/5QP36).

## Ethics Statement

The animal study was reviewed and approved by King's College London Animal Welfare and Ethical Review Body.

## Author Contributions

This work was conceptualized by EM and DC as a secondary analysis in a study designed by DC and FT. EM, PW, and OE designed the resource generation. EM, CS, EK, and DC collected the data. EM, EK, and IB contributed to image processing. CS and DC performed the histological quantification. EM and FT designed the statistical analysis. EM performed the statistical analysis and wrote the first draft of the manuscript. All authors contributed to manuscript revision, read, and approved the submitted version.

## Funding

The study was funded by the UK Biotechnology and Biological Sciences Research Council [BB/N009088/1]. EM was supported by the UK Medical Research Council [MR/N013700/1] and King's College London as a member of the MRC Doctoral Training Partnership in Biomedical Sciences.

## Conflict of Interest

The authors declare that the research was conducted in the absence of any commercial or financial relationships that could be construed as a potential conflict of interest.

## Publisher's Note

All claims expressed in this article are solely those of the authors and do not necessarily represent those of their affiliated organizations, or those of the publisher, the editors and the reviewers. Any product that may be evaluated in this article, or claim that may be made by its manufacturer, is not guaranteed or endorsed by the publisher.

## Abbreviations

MRI: magnetic resonance imaging
T1w: *T*_1_-weighted
T2w: *T*_2_-weighted
TIV: total intracranial volume
GM: gray matter
GMV: gray matter volume
WM: white matter
CSF: cerebrospinal fluid
TPM: Tissue Probability Map
EMM: Estimated Marginal Mean.
